# Metamorphopsia Score and Central Visual Field Outcomes in Diabetic Cystoid Macular Edema

**DOI:** 10.1155/2018/4954532

**Published:** 2018-03-18

**Authors:** Agnieszka Kalinowska, Katarzyna Nowomiejska, Agnieszka Brzozowska, Ryszard Maciejewski, Robert Rejdak

**Affiliations:** ^1^Department of General Ophthalmology, Medical University, Lublin, Poland; ^2^Department of Mathematics and Medical Biostatistics, Medical University, Lublin, Poland; ^3^Human Anatomy Department, Medical University, Lublin, Poland; ^4^Department of Experimental Pharmacology, Medical Research Centre, Polish Academy of Sciences, Warsaw, Poland

## Abstract

**Aim:**

To detect abnormality of the visual function in naïve patients with cystoid diabetic macular edema (DME) using M-charts, Amsler test, and white on white (W/W) and blue on yellow (B/Y) perimetry.

**Methods:**

There were 64 eyes included in the study: 30 eyes with DME, 22 eyes with diabetes without DME, and 12 eyes of normal subjects. Conventional W/W perimetry and B/Y perimetry were performed within the central 10° of the visual field. To assess metamorphopsia, Amsler test and M-charts were used.

**Results:**

The rate of detection of metamorphopsia was 37% with Amsler test examination and 50% with M-charts. Specificity of both tests was 100%. We found a significant difference between vertical scores of M-charts in all groups, but not in horizontal scores (*p* < 0.0001). Mean defect (MD) was 8.9 dB and 3.6 dB and loss variance (LV) 4.8 dB and 3.3 dB (*p* < 0.0001).

**Conclusions:**

M-chart is more sensitive than Amsler test method for detection of metamorphopsia. The MD and LV are higher in b/y in comparison to W/W perimetry. B/Y perimetry and M-charts are more sensitive than conventional methods for detecting the visual function loss in cystoid DME.

## 1. Introduction

Diabetes mellitus is one of the most common chronic global diseases. The global prevalence of diabetes among adults is expected to be increased to 7.7% of population, 439 million people by 2030 [1]. Diabetic macular edema (DME) is a complication of diabetic retinopathy and is the most common cause of the visual loss in both proliferative and nonproliferative diabetic retinopathy. Approximately 20% of the patients with diabetic retinopathy are affected by macular edema [2].

There are different patterns of fluid accumulation in DME [3]. Cystoid DME is an accumulation of fluid in the cystoid spaces of the outer plexiform layer of the retina resulting in increased retinal thickness. It is due to a leakage from microaneurysms and retinal capillaries due to extensive breakdown of the blood-retinal barrier, release of various cytokines, and significant inflammation [4]. Other patterns include diffuse retinal thickening and serous retinal detachment; those patterns are hallmarks of chronic macular edema. Because of these processes the normal retinal architecture is damaged and results in a loss of visual acuity, central scotoma, and metamorphopsia.

Visual acuity is a parameter commonly used in clinical practice to evaluate the visual function in DME but it does not always provide a complete estimate of the visual abilities [5]. Metamorphopsia is one of the most important symptoms occurring in eyes with macular diseases; it appeared if the photoreceptors and their outer segments are displaced from their origin positon [6]. The Amsler grid is a gold standard for detecting metamorphopsia and is widely used since 1947 [7]. However it is impossible to quantify the severity of metamorphopsia using only Amsler grid. M-charts, invented by Matsumoto et al. [8], are very a cheap, easy, and safe clinical method to define the degree of metamorphopsia; that is why it can be useful in monitoring patients with DME.

It has been already shown that perimetry can provide more useful information than visual acuity on functional loss in diabetic retinopathy, particularly when the perifoveal capillary network is damaged [9]. Short Wavelength Automated Perimetry (SWAP) utilizes a blue stimulus to preferentially stimulate the blue cones and yellow background to adapt the green and red cones and to saturate, simultaneously, the activity of the rods. This method is established in early detection of glaucoma [10], and it detects changes at the retinal ganglion cell level and loss of retinal nerve fibers. It has also been used in patients with diseases as retinitis pigmentosa and diabetic retinopathy [11–14], where changes are located in the inner retina. The principle of the short wavelength sensitive (SWS) cone-mediated mechanisms is that the blue cones are more susceptible to damage in diabetes [15]. That is why SWAP may be an early detector of visual function loss in diabetic maculopathy [16].

The aim of this study was (1) to assess metamorphopsia using Amsler test and M-charts, (2) to detect abnormality of the central visual field using white on white (W/W) and blue on yellow (B/Y) perimetry in patients with DME, and (3) to compare the results with the best corrected visual acuity and OCT examination.

## 2. Methods

There were fifty-two patients prospectively recruited from the retinal outpatient clinic at the Department of General Ophthalmology in Lublin, Poland. The study was performed in accordance with the Declaration of Helsinki. Written informed consent was taken from each participant after explaining the nature of the study. The study was approved by the local Ethics Committee. The study excluded those who had a history of glaucoma, opaque media, and refractive error greater than three-dimensional (3D) spherical equivalent, as well as patients with best corrected visual acuity (BCVA) less than 0.5 and patients with preretinal or vitreous hemorrhage, epiretinal membrane, macular hole, vitreoretinal traction, previous laser therapy, or anti-VEGF injections.

Patients were divided into two groups. Group 1 consisted of 30 eyes with newly diagnosed cystoid DME due to type II diabetes (14 males, 16 females) with mean age of 61 years (range: 35–78 years). Group 2 consisted of 22 eyes with type II diabetes and without DME (7 males, 15 females), with mean age of 60 years (range: 35–71 years). Additionally, 12 eyes of healthy probands (2 males, 10 females), with mean age of 57 years (range: 51–62 years), served as a control group. Complete ophthalmological examination with BCVA measurements (Snellen charts), slit lamp examination, applanation tonometry, dilated funduscopy, and OCT were performed on each eye. The central retinal thickness (CRT) measurements were performed with three-dimensional (3D) OCT-2000 (Topcon Corporation, Tokyo, Japan). The OCT was conducted by the same investigator (AK) in all patients. Each eye was examined after pupillary dilatation. Macular 3D scan protocol (6 mm × 6 mm area centered on the fovea with a scan density of 512 [vertical] × 128 [horizontal] scans) was used for all patients. To provide more precise OCT details we obtained mean retinal thickness measurements in 9 Early Treatment Diabetic Retinopathy Study (ETDRS) regions. The regions were located in three rings with radius of 1, 3, and 6 mm around the fovea. The rings were segmented into 4 quadrants (S: superior, I: inferior, N: nasal, and T: temporal). To make it easier to discuss this, we used shortcuts to label each region; for example, I3 defines inferior subfield in 3 mm ring and T6 temporal subfield in 6 mm ring. The central 1 mm ring was labelled C1 the Central Retinal Subfield (Figure 10). Each OCT scan was analysed using the onboard Topcon's 3DOCT software. It defines the inner and outer retinal boundaries as the internal limiting membrane (ILM) and the inner boundary of the retinal pigment epithelium (RPE). The automated measurements (in *μ*m) of retinal thickness in each of the 9 ETDRS subfields were recorded.

In the first group we included diabetic patients only with cystoid macular edema, not diffuse nor serous retinal detachment [3]. Macular edema was defined on the basis of OCT as the presence of intraretinal cysts in the outer plexiform layer without disruption of ellipsoid zone and without subretinal fluid and epiretinal membrane or vitreomacular traction.

### 2.1. Visual Function Assessment

Visual function testing included BCVA using decimal Snellen charts, standard Amsler chart (black grid on a white background), vertical and horizontal M-charts with near correction, and undilated pupils, as well as white on white (W/W) and blue on yellow (B/Y) perimetry inside 10 degrees of the visual field. The Amsler grid test examination was done for all patients in the same examination room with the same lighting conditions. All the patients were examined by the same person (physician) after clear instructions. The examination was performed at the distance of 30 cm with appropriate near correction. An eye patch occluded the eye not being tested, and the patients were instructed to fixate on the central point of the grid at all times. The M-charts were used to assess both the horizontal and vertical metamorphopsia scores. The M-charts consist of 19 dotted (dot size is 0.1°) lines with dot intervals ranging from 0.2° to 2.0° of visual angle. In the center of each line, there is a fixation point of 0.3°. The examination is performed at the distance of 30 cm with appropriate near correction. Dotted lines with interval changes from fine to coarse are printed on the following paper pages and are shown to the patients one after another. The examiner presents consecutive dotted lines, starting with a solid line (0°) and the patient has to state whether the presented line is distorted or not. If the patient recognizes the dotted line as being straight, its visual angle is considered to be the metamorphopsia score. M-charts are presented in vertical and horizontal direction after rotating of 180°.

We analysed only the second perimetry examination; the first was done only for patients perimetry training. The patients were given very clear instructions. In the first step “white on white” perimetry was performed and afterwards “blue on yellow” perimetry. Perimetry was performed using macula M program with TOP strategy on Octopus 900 perimeter (Haag-Streit, Switzerland) with both W/W (target size III) and B/Y (target size V) stimuli. The patient's correction was adjusted for a viewing distance of 30 cm. The visual field charts were reviewed for mean deviation (MD) and loss variance (LV).

### 2.2. Statistical Analysis

Statistical analysis was performed using STATISTICA 13.0 software (StatSoft, Krakow, Poland). All values are presented as the means ± standard deviation. The Amsler test was considered positive if any blurred lines were reported by the patient. The M-chart result was considered positive if the metamorphopsia score was more than 0° (0.2–2.0°). If it was 0°, it was considered negative. This assessment was done in regard to vertical and horizontal charts separately. The Kruskal–Wallis test was used to compare many independent groups; bivariate relationships were analysed using Pearson's correlation coefficient comparisons of means. *p* < 0.05 indicated the statistical significance.

## 3. Results

### 3.1. Visual Acuity

The mean BCVA (Snellen) differed significantly between eyes of the healthy control group (1.0 ± 0.0), eyes of diabetic patients without DME (0.94 ± 0.1), and eyes of diabetic patients with cystoid DME (0.75 ± 0.20) (*p* < 0.0001). Pearson's correlation coefficient pairwise comparisons of means indicated that the mean BCVA values in the eyes of the healthy control group and the diabetic patients with no DME did not differ significantly (*p* = 0.67). On the other hand, mean BCVA value in the eyes of the diabetic patients with cystoid DME was significantly worse than that in the eyes of the healthy control group (*p* = 0.0003) and the diabetic patients with no DME (*p* = 0.004).

The mean near visual acuity was significantly worse in group with macular edema (0.57 ± 017), than that in healthy eyes (0.5 ± 0,0) or eyes without edema (0.5 ± 0.0) (*p* = 0.05); but using Pearson's correlation coefficient, there were no significant differences between all groups (*p* > 0.05). There was no correlation found between BCVA and CRT in OCT examination (*p* > 0.05).

### 3.2. Central Retinal Thickness

The morphological changes of the retina in DME have been assessed quantitatively using OCT. The central retinal thickness (CRT) was defined as a mean thickness of the neurosensory retina (between internal limiting membrane and retinal pigment epithelium) in the central 1 mm diameter regions measured using OCT mapping software. The mean CRT in OCT differed significantly between DME group (293.97 *μ*m ± 68.17 *μ*m) and the group with diabetes and without DME (243.59 *μ*m ± 28.31 *μ*m) (*p* = 0.001); there were no significant differences between the group with diabetes and without DME and the control group (250.08 *μ*m ± 12.01 *μ*m) (*p* = 1.00) (Figure 3). It should be noted that in all groups superior zone in 3 mm is the thickest and temporal zone in 6 mm is the thinnest part of the macula (Table 1). We calculated Pearson correlation coefficients to determine whether CRT is associated with changes in macular thickness in ETDRS regions. The statistical analysis shows that there is a strong correlation between CRT and retinal thickness in I3 (*p* = 0.003) and N6 (*p* = 0.002) regions in group with macular edema. An increase in the CRT value influences the increase of these retinal thickness values. There was no significant correlation between CRT and other remaining values of retinal thickness (*p* > 0.05) in all groups.

### 3.3. Metamorphopsia Scores

**Figure 1:**
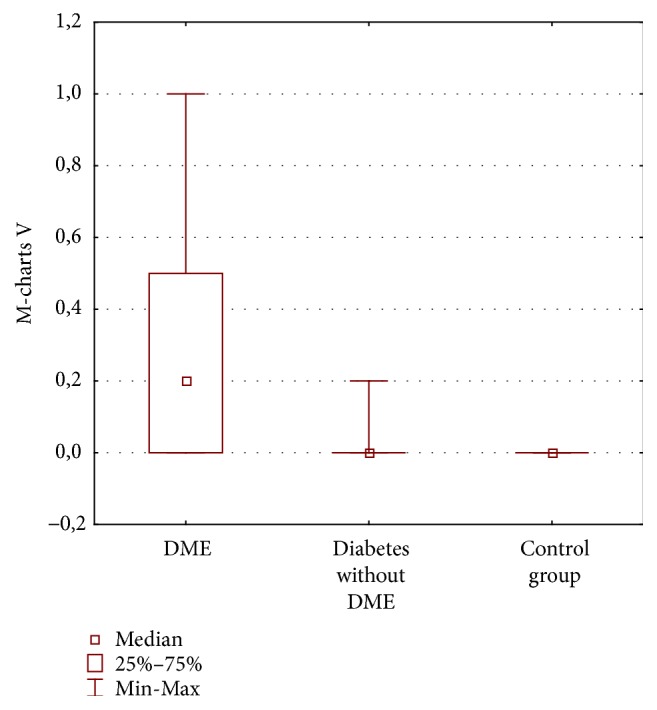
Vertical (V) M-charts scores (degrees) in three groups of patients: diabetic macular edema (DME), diabetes without DME, and control group.

**Figure 2:**
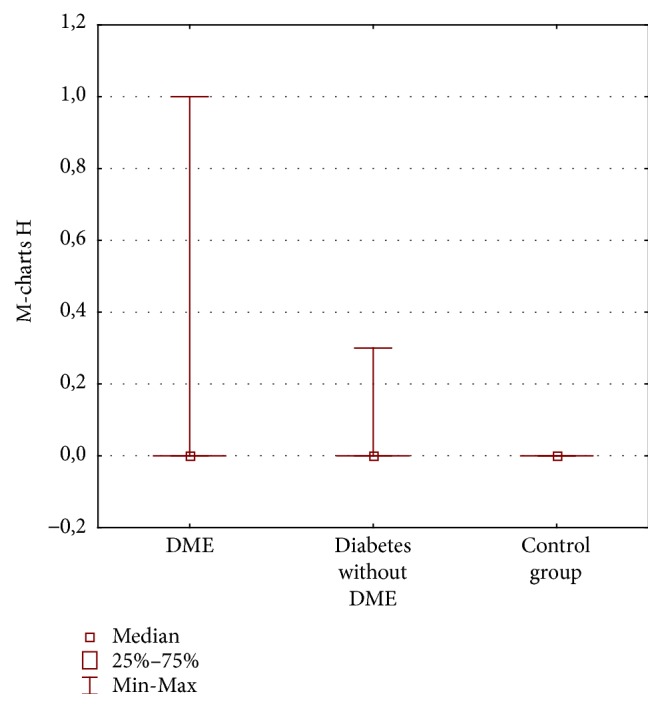
Horizontal (H) M-charts scores (degrees) in three groups of patients: diabetic macular edema (DME), diabetes without DME, and control group.

**Figure 3:**
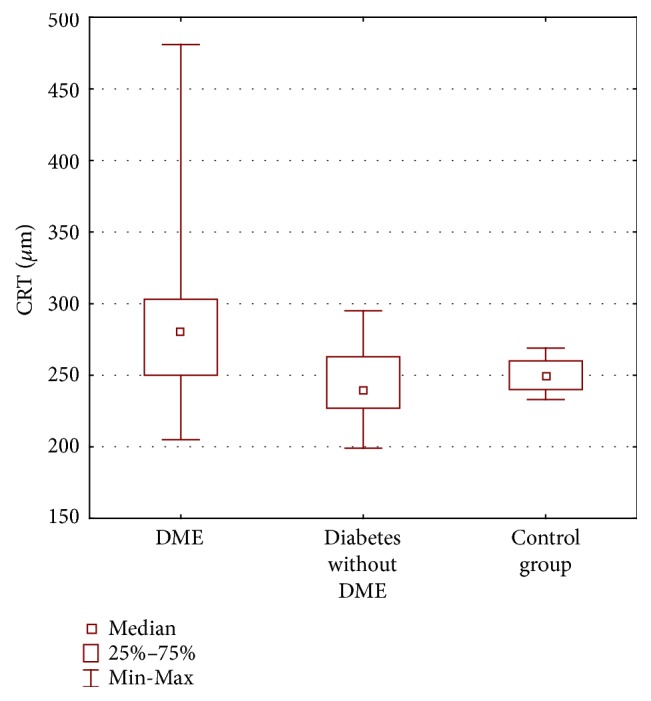
Central retinal thickness (CRT) in micrometers (*μ*m) obtained by ocular coherence tomography examination in three groups of patients: diabetic macular edema (DME), diabetes without DME, and control group.

The mean vertical and horizontal scores in the group of eyes with DME were 0.25°  ±  0.27° and 0.14°  ±  0.27°, respectively, and in the group with diabetes and without DME they were 0.01°  ±  0.04° and 0.03 ± 0.09, and in the control group no metamorphopsia was detected (0° score in M-charts) (Figures 1 and 2). We found no significant difference analysing H-scores between all three groups (*p* = 0.08), but there was a significant difference between V-scores in all groups (*p* < 0.0001). Pearson's correlation coefficient showed significant difference between DME group and group with diabetes and without DME (*p* = 0.003) and also between Group 1 and control group (*p* = 0.01) but detected no differences between group without DME and control group.

Healthy eyes in the control group revealed no metamorphopsia both with Amsler test and M-charts.

Amsler test was abnormal in 37% in the DME group: it was normal in 100% of the control group and in the group with diabetes without DME (*p* = 0.0003). Vertical M-charts were abnormal in 57% of DME eyes and in 5% of diabetic patients without DME and were normal in 100% of the control group (*p* = 0.00001). Horizontal M-charts were abnormal in 23% of DME eyes and in 9% of diabetic patients without DME and were normal in 100% of the control group (*p* = 0.1).

### 3.4. Perimetry

The mean MD in DME group was 5.44 dB ± 3.30 dB and it significantly differed from group with diabetes and without DME (2.77 dB ± 2,13 dB) (*p* = 0.007); there was no difference between group with diabetes without DME and control group (2.99 dB ± 2.33 dB) (*p* = 1.00). The mean MD in w/w perimetry significantly differed between all three groups (*p* = 0.005) (Figure 6).

The mean LV in w/w perimetry also differed significantly between all the groups (*p* < 0.0001). It was 3.45 dB ± 2.08 dB in DME group, 2.39 dB ± 0.62 dB in a group with diabetes without DME, and 1.43 dB ± 0.44 dB in a control group. It was significantly different between DME group and control group (*p* = 0.00001) as well as between group with diabetes and without DME and control group (Figure 7).

**Figure 4:**
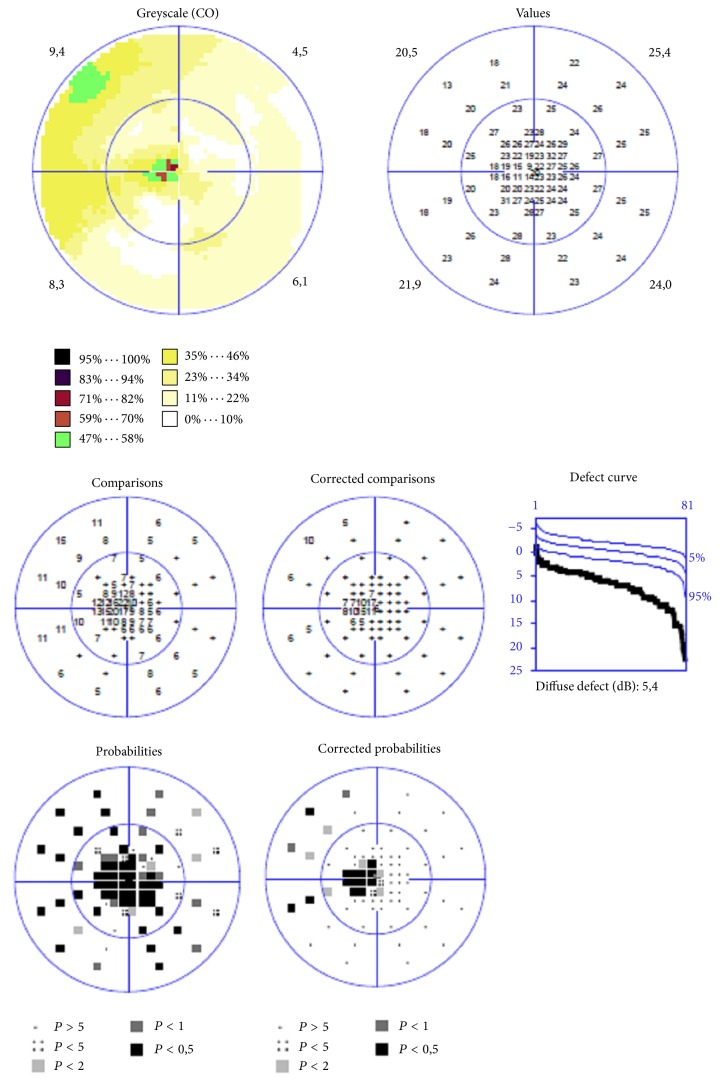
Printout of the visual field examination, “white on white” static automated perimetry (Octopus 900) of the patient with diabetic macular edema, small central scotoma.

Representative case of W/W perimetry in DME patient is shown in Figure 4.

The mean MD in SWAP perimetry significantly differed between all the groups (*p* < 0.0001). The mean MD in group with DME was 9.20 dB ± 4.23 dB; in group without DME it was 4.47 dB ± 3.38 dB (*p* = 0.00006); there was no differences between group with diabetes without DME and control group (4.68 dB ± 1.26 dB) (*p* = 1.00) (Figure 8).

The mean LV in SWAP perimetry also differed significantly between all the groups (*p* < 0.0001). It was significantly different between Group 1 and Group 2 (*p* = 0.00005) as well as between Group 1 and control group (*p* = 0.000003) but there was no significant difference between Group 2 and control group (*p* = 0.55).

**Figure 5:**
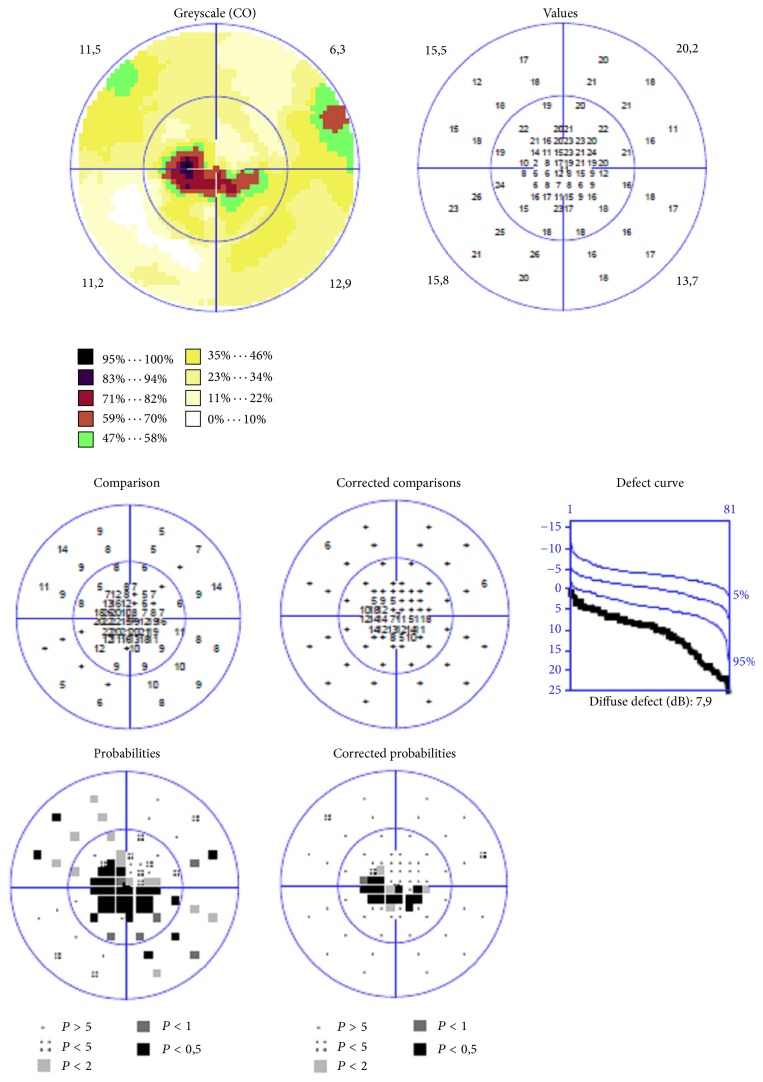
Printout of the visual field examination “blue on yellow” static automated perimetry (Octopus 900) of the patient with diabetic macular edema, large central scotoma.

**Figure 6:**
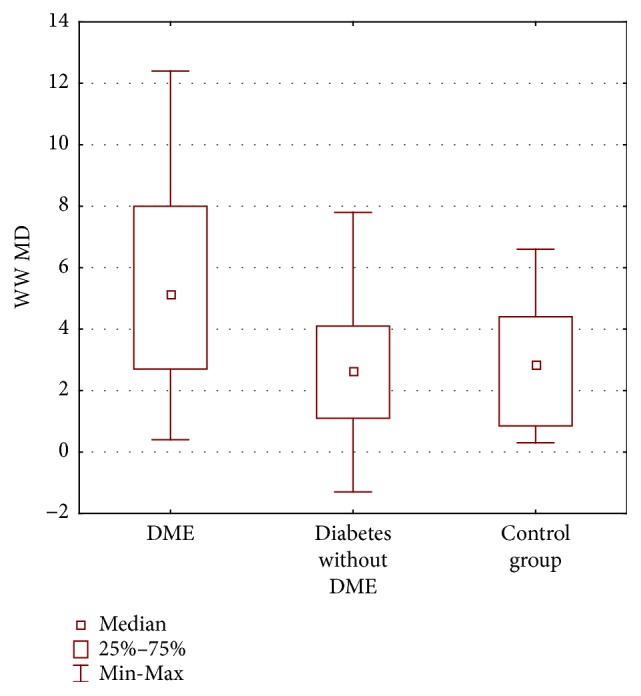
Mean deviation (MD) in decibels (dB) W/W perimetry in three groups of patients: diabetic macular edema (DME), diabetes without DME, and control group.

**Figure 7:**
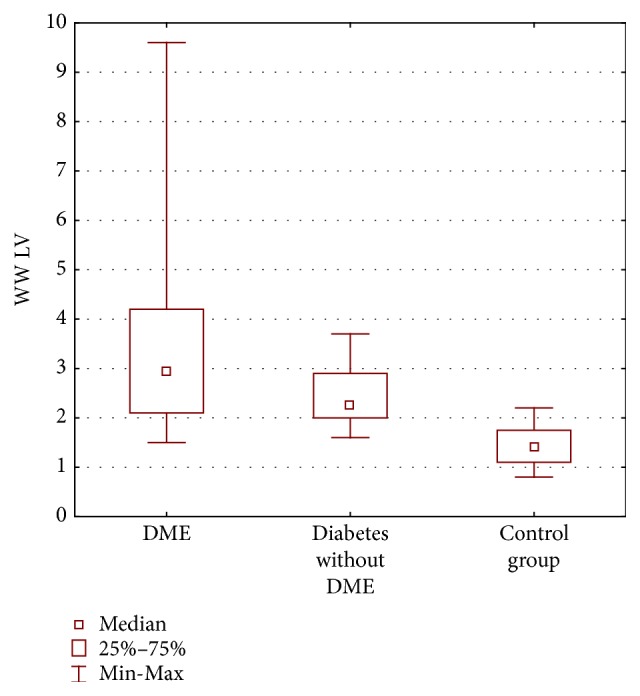
Loss variance (LV) in decibels (dB) w/w perimetry in three groups of patients: diabetic macular edema (DME), diabetes without DME, and control group.

**Figure 8:**
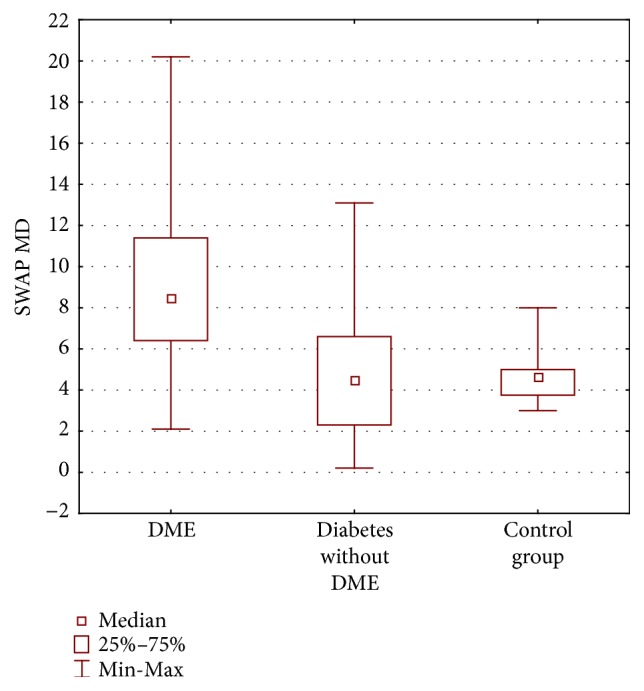
Mean deviation (MD) in decibels (dB) SWAP perimetry in three groups of patients: diabetic macular edema (DME), diabetes without DME, and control group.

Representative case of SWAP perimetry in DME patient is shown in Figure 5.

**Figure 9:**
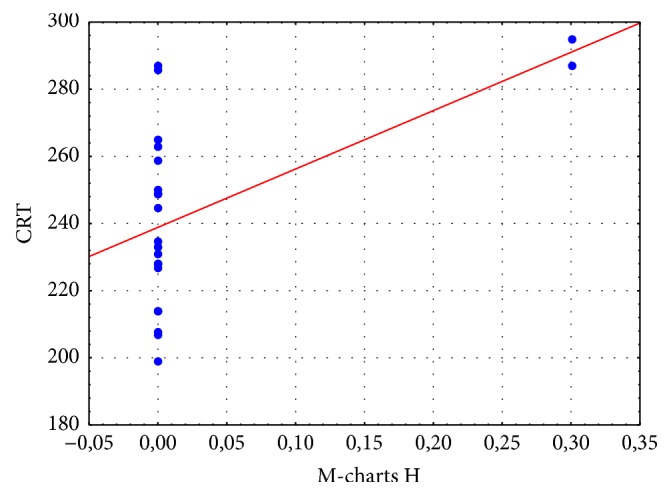
Correlation between horizontal M-charts and (M-charts H) and central retinal thickness (CRT) in micrometers in patients with diabetic macular edema. Correlation coefficient (red line) *R* = 0.49. There is an increase of M-charts H with increasing CRT.

**Figure 10:**
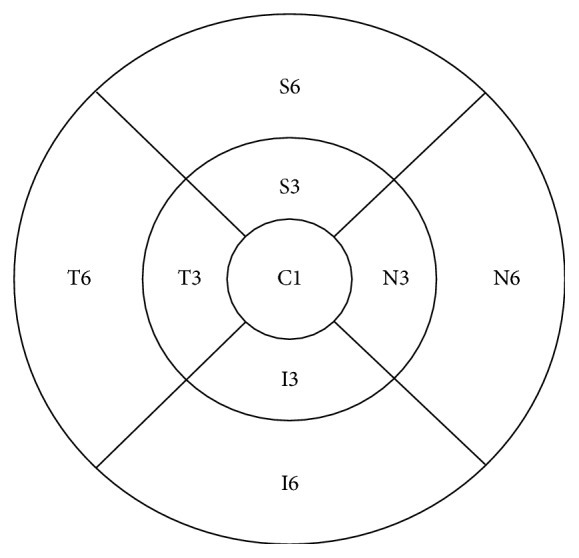
Nine Early Treatment Diabetic Retinopathy Study (ETDRS) regions, shortcuts (T: temporal, S: superior, I: inferior, and N: nasal, each 3 or 6 mm from the fovea).

There was a positive correlation found between horizontal M-charts and CRT in OCT (*R* = 0.49, *p* = 0.02). There was no correlation found between horizontal M-charts and other parameters (Figure 9).

## 4. Discussion

The present study is the first prospective research for detecting metamorphopsia in cystoid DME eyes using M-charts and comparing the results with diabetes patients without macular edema and control group. We demonstrated that the significant number of patients with DME suffered from some visual field disorders and 50% of them have positive M-charts test. The patients were very precisely selected; in all groups the age was similar; BCVA was better than 0.5 and no previous treatment was detected. The sensitivity of Amsler test was only 37,5% but it has been already reported that Amsler grid has very poor rate of detecting metamorphopsia. The sensitivity was better when M-charts were used and it was 50%. M-charts have been already used in patients with epiretinal membranes [17–19], macular holes [20], branch retinal vein occlusion [21], and age related macular degeneration [22].

Achiron and coworkers [17] examined with M-charts 15 eyes of 10 patients with DME. They observed positive M-charts score in 46.6% of eyes, which is similar to our results. In the study of 36 AMD patients the rate of metamorphopsia detection was 89% with M-charts and 69% with Amsler test [22]; thus it was higher than in DME eyes.

In our study we have found significant differences between groups (DME versus non-DME) only in vertical metamorphopsia scores. This aspect was not analysed in the study of Achiron. The difference between horizontal and vertical metamorphopsia has been already observed by Amsler [23] and Matsumoto et al. [18]. In a study of Nowomiejska and coworkers it has been found that the decrease of metamorphopsia after intravitreal injection of anti-VEGF in AMD patients was significant for horizontal lines only [22]. In our study we included patients with BCVA better than 0.5 according to Snellen. Thus, they represent cystoid macular edema that may be localized to quadrants around the fovea. We have found that the retina was the thickest in the superior quadrant from the fovea, so the macular edema was the most prominent in the vertical line. We have also found that there was significant difference among V-scores in all groups using M-charts. This is similar to conditions reported by Murakami and colleagues [24] in evaluation of metamorphopsia in patients with branch retinal vein occlusion (BRVO). They hypothesized that because the lesion is located either above or below the fovea, the detection power tends to be stronger for vertical lines than for horizontal lines on M-charts. What is more, in the recent report on eyes with BRVO and central retinal vein occlusion (CRVO) by Manabe and coworkers [25], they observed that the mean total foveal thickness was higher in eyes with CRVO and the severity of macular edema seems to be greater, but the prevalence and degree of metamorphopsia were unexpectedly higher in BRVO. The advantage of M-charts is that they are easy, cheap, and simple to use. They can be used as an another visual function indicator in patients with maculopathies of different origin and also in DME. Moreover, application of M-charts may lead to a better care of diabetic patient [26].

As an improvement of traditional Amsler test, specialized three-dimensional computer-automated threshold Amsler grid testing (3D-CTAG) has been evolved [27] and applied in 16 patients with DME and 21 with AMD. Amsler grid testing was performed in 16% of subjects. All eyes showed significant increases in CVF deficit surface area at minimum contrast levels when compared to maximum contrast.

Nowadays the gold standard for diagnosing macular edema is OCT, as it gives very detailed information about morphological changes in macula; however it does not provide any knowledge in regard to the visual function.

Visual acuity loss is well known in patients with DME but it always appears in the moment when edema affects the center of the macula. That is why the visual function test that could identify macular edema before the BCVA is affected would be great value.

Neurodegeneration is an early component of diabetic retinopathy. It is unclear whether neurodegeneration is an independent factor or a consequence of damaged retinal vasculature [28]. Hyperglycemia disrupts the delicate metabolic environment in the retina and reduced signals from the insulin receptor, which are essential for neuronal development.

Growth and survival lead to neural apoptosis [29]. The presence of selective loss of SWS pathways in diabetic patients has been known for many years. Firstly it was detected in laboratory study using foveal stimuli [30–32]. However the pioneers in using SWAP to investigate DME were Lutze and Bresnick [13]. In 1998 they evaluated SWAP and W/W perimetry in 24 diabetes patients with clinically significant macular edema. All 24 patients exhibited focal defects for SWAP compared to only one-third for W/W perimetry. The topography of the SWAP defect corresponded to the clinical mapping of the area of DME.

Remky and coworkers examined 45 patients with diabetes without macular edema [16]; SWAP and WWP within 10° were performed. Analysis revealed significantly lower sensitivity in diabetes patients than in controls. The findings of Remky and colleagues together with those of Hudson and colleagues suggest that SWAP may have an application in the detection of ischaemic damage of the macula and of those patients at risk of developing DME.

SWAP has already been compared to SAP in diabetic patients with and without diabetic retinopathy [33]. The limitation of SWAP in diabetic patients is reduced transmission of the short wavelength stimulus arising from ocular media absorption (cataract); thus we excluded patients with cataract [34].

In our study we observed more pronounced visual field central defects obtained with SWAP than with w/w perimetry. This suggests that SWS sensitivity may be affected in patients with cystoid DME and diabetic patients without DME. SWAP is considered an earlier indicator of function loss in ischaemic change in diabetic retinopathy than SAP. There are both significant overall sensitivity reduction and localized defects, more severe in DME and diabetic patients without DME.

The limitation of our study is that we have not used microperimetry to assess the sensitivity of the retina in the macula region. The larger cohort of patients with cystoid DME may be examined in the future; we can also consider a follow-up of visual function assessment in DME patients, as well as effectiveness of anti-VEGF treatment.

In conclusion, this study assessed prospectively metamorphopsia using M-charts and Amsler test and visual field outcomes of W/W and B/Y perimetry in DME patients. Both M-charts and B/Y perimetry are more sensitive than traditional methods. They provide additional diagnostic tools to assess the visual function of patients with cystoid DME.

## Figures and Tables

**Table 1:** Evaluation of retinal thickness in micrometers in ETDRS regions around the fovea. *Abbreviations*. DME-diabetic macular edema, S3: superior quadrant inside 3 mm around the fovea, S6: superior quadrant inside 6 mm around the fovea, I3: inferior quadrant 3 mm around the fovea, I6: inferior quadrant 6 mm around the fovea, T3: temporal quadrant 3 mm around the fovea, T6: temporal quadrant 6 mm around the fovea, N3: nasal quadrant 3 mm around the fovea, and N6: nasal quadrant 6 mm around the fovea.

	DME			Diabetes without DME			Control group			*p*
	Mean	Median	Std. deviation	Mean	Median	Std. deviation	Mean	Median	Std. deviation	
S3	327.25	318.00	60.22	291.38	293.00	17.60	317.92	326.00	15.83	0.008
S6	291.05	274.50	55.58	251.62	252.00	14.13	280.42	280.50	9.78	0.0004
I3	319.85	306.50	48.75	288.31	283.00	12.97	314.92	318.00	13.37	0.004
I6	279.50	273.00	37.13	255.54	251.00	11.60	267.75	267.50	12.28	0.03
T3	312.95	297.00	52.37	284.46	282.00	13.45	303.25	303.00	13.59	0.03
T6	283.50	265.50	40.39	246.69	247.00	11.89	262.25	264,50	10.08	0.004
N3	318.10	310.50	32.00	292.08	291.00	13.59	315.58	319.00	12.31	0.002
N6	289.80	284.50	25.79	268.08	271.00	11.39	294.83	291.50	13.52	0.0002
